# High‐Performance Hot‐Exciton OLEDs via Fully Harvesting Triplet Excited States from Both the Exciplex Co‐Host and the TBRb Emitter

**DOI:** 10.1002/advs.202303192

**Published:** 2023-08-16

**Authors:** Fuxian Wei, Jing Chen, Xi Zhao, Yuting Wu, Huiyao Wang, Xiaoli Chen, Zuhong Xiong

**Affiliations:** ^1^ Chongqing Key Laboratory of Micro & Nano Structure Optoelectronics, School of Physical Science and Technology Southwest University Chongqing 400715 P. R. China

**Keywords:** dexter energy transfer, hot exciton, magneto‐electroluminescence, organic light‐emitting diode, reverse intersystem crossing

## Abstract

The high‐level reverse intersystem crossing (HL‐RISC, T_2_ → S_1_) process from triplet to singlet exciton, namely the “hot exciton” channel, has recently been demonstrated in the traditional fluorescent emitter of TBRb. Although it is a potential pathway to improve the utilization of non‐radiative triplet exciton energy, highly efficient fluorescent organic light emitting diodes (FOLEDs) based on this “hot exciton” channel have not been developed. Herein, high‐efficiency and low‐efficiency roll‐off FOLEDs are achieved through doping TBRb molecules into an energy‐level matched exciplex co‐host. Combining the low‐level RISC (LL‐RISC, EX_3_ → EX_1_) process in the exciplex co‐host with the HL‐RISC process of hot excitons in TBRb to fully harvest the triplet energy, a record‐high external quantum efficiency (EQE) of 20.4% is obtained via a proper Dexter energy transfer of triplet excitons, realizing the efficiency breakthrough from fully fluorescent material‐based OLEDs with TBRb as an end emitter. Furthermore, the fingerprint Magneto‐electroluminescence (MEL) as a sensitive measuring tool is employed to visualize the “hot exciton” channel in TBRb, which also directly verifies the effective energy confinement and the full utilization of hot excitons. Obviously, this work paves a promising way for further fabricating high‐efficiency TBRb‐based FOLEDs for lighting and flat‐panel display applications.

## Graphical abstract

A high‐performance hot exciton‐type organic light‐emitting diode with an external quantum efficiency of over 20% is successfully obtained. It is attributed to the synergistic effect of the low‐level reverse intersystem crossing (RISC, EX_1_←EX_3_) of exciplex states in the exciplex co‐host and the high‐level RISC (S_1_←T_2_) channels of hot excitons in the TBRb guest.

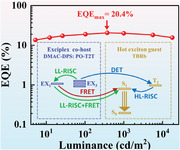

## 1 Introduction

In recent years, TBRb (2,8‐Di‐tert‐butyl‐5,11‐bis(4‐tert‐butylphenyl)−6,12diphenyltetracene) as a fluorescent emitter has been widely employed to design high‐efficiency organic light‐emitting diodes (OLEDs) for its excellent advantages of large photoluminescence quantum yield, high radiation decay rate, and high color purity.^[^ ^1^ ^]^ In 2014, *Adachi* and coworkers prepared a hyperfluorescence‐based fluorescent OLED (FOLED) by co‐doping a thermally activated delayed fluorescence (TADF) sensitizer and the TBRb emitter in a host matrix. In this device, an external quantum efficiency (EQE) of 18.0% was achieved by utilizing a reverse intersystem crossing (RISC) process from triplet to singlet in the TADF sensitizer to harvest its non‐radiative triplet state energy and then transferring the singlet state energy to the end emitter of TBRb.^[^ ^2^ ^]^ In 2019, Dongge Ma et al. developed a highly efficient yellow FOLED with an EQE of 17.0% through the phosphor sensitization approach to sensitize the fluorescent emitter of TBRb.^[^ ^3^ ^]^ In 2020, Xialei Lv et al. designed a high‐efficiency FOLED by combining dual TADF hosts and the TBRb emitter, and the maximum EQE value of this kind of FOLED reached 19.1%.^[^ ^4^ ^]^ Although researchers have consistently improved the device efficiency by optimizing the device structure in recent years, the energy loss problem of the non‐radiative triplet states remains a major impediment to further enhancing the device's performance. Specifically, the low‐level Dexter energy transfer (DET, T_1, host_ → T_1, TBRb_) process from the triplet state of the host or sensitizer to the first excited triplet state of the TBRb emitter is a main energy loss channel that is detrimental to the light emission due to the non‐radiative decay from T_1, TBRb_ to the ground state.^[^ ^5^ ^]^ Therefore, a significative question is whether the emission efficiency of TBRb‐based devices can be further improved by avoiding the generation of non‐radiative T_1, TBRb_ excitons.

As reported in the literature, a small singlet‐triplet energy splitting $\left(\Delta E_{S_{m}T_{n}}\right)$ between singlet exciton of S_m_ and high‐lying triplet exciton of T_n_ in an organic semiconducting molecule will motivate a fast high‐level RISC process from T_n_ to S_m_ states (HL‐RISC, T_n_ → S_m_, n≥ 2, m≥ 1), namely the “hot exciton” mechanism.^[^ ^6^ ^]^ Yuguang Ma et al. have developed many efficient blue emission materials based on the “hot exciton” channel.^[^ ^7^ ^]^ In our recent work, we found that the HL‐RISC channel (T_2, TBRb_ → S_1, TBRb_) also exists in the conventional fluorescent emitter of TBRb $\left(\Delta \;E_{S_{1}T_{2}}=\;-0.11\;\mathrm{eV}\right)$.^[^ ^8^ ^]^ That is, once the T_2, TBRb_ state is generated, it will be readily converted to the radiative S_1, TBRb_ state through the fast HL‐RISC process rather than being lost via the non‐radiative decay pathways provided that the triplet exciton energies in host and charge‐transport layers are higher than T_2, TBRb_ excitons (Namely, T_2, TBRb_ excitons are well confined in the device emitting layer). Obviously, this provides a new idea for designing high‐efficiency FOLEDs through high‐level DET process (HL‐DET, T_1, host_ → T_2, TBRb_) to transfer the host triplet energy to the T_2, TBRb_ state instead of the T_1, TBRb_ state because the internal conversion (IC) channel of hot triplet excitons in TBRb (T_2, TBRb_ → T_1, TBRb_) could be negligible due to their large energy difference ($\Delta E_{T_{2}T_{1}}\;$> 1.0 eV).^[^ ^9^ ^]^ In this case, the energy loss resulting from the non‐radiative T_1, TBRb_ state can be successfully prevented.

In this work, fully fluorescent material‐based OLEDs with high‐efficiency and low‐efficiency roll‐off were obtained by dispersing the TBRb dopant molecules into an energy level‐matched exciplex co‐host as the emitting layers (EMLs). Here, 10,10′‐(4,4′‐Sulfonylbis(4,1‐phenylene))bis(9,9dimethy‐9,10‐di‐hydroacridine (DMAC‐DPS) and 2,4,6‐Tris[3‐(diphenylphosphinyl)phenyl]−1,3,5‐triazine (PO‐T2T) were selected as donor and acceptor to form the exciplex co‐host in a 1:5 weight ratio, and TBRb molecules were simultaneously doped into this mixing system with different doping concentrations (0.5, 1, 3, and 5 wt.%). A maximum EQE of 20.4% was successfully achieved in the 0.5 wt.% TBRb‐doped FOLED. To the best of our knowledge, this EQE is the highest value among the previously reported best performance OLEDs made from fully fluorescent materials with TBRb as an end emitter and without any sensitizer.^[^ ^1^, ^5^ ^]^ This achievement in both high‐efficiency and low‐efficiency roll‐off is mainly attributed to the simultaneous utilization of the low‐level RISC process (LL‐RISC, EX_3, co‐host_ → EX_1, co‐host_) in the exciplex co‐host and the HL‐RISC process (T_2, TBRb_ → S_1, TBRb_) of hot excitons in the TBRb emitter to fully harvest the nonradiative triplet states. In particular, the efficient HL‐DET process and negligible low‐level DET process ensure the sustained generation of the T_2, TBRb_ states instead of the unexpected T_1, TBRb_ states, thus the exciton energy loss is well avoided. Moreover, the sensitive probing tool of fingerprint Magneto‐electroluminescence (MEL) was employed to further visualize the underlying physical mechanisms in these high‐performance FOLEDs. Accordingly, this work not only enriches the physical understanding of the microscopic evolution channels in TBRb, but also provides a new reference for designing high‐performance TBRb‐based OLEDs for lighting applications and flat‐panel displays.

## 2 Results and Discussion

### 2.1 Energy Transfer Processes in TBRb‐Doped FOLEDs and Their Optical‐Electrical Performances

**Figure 1:**
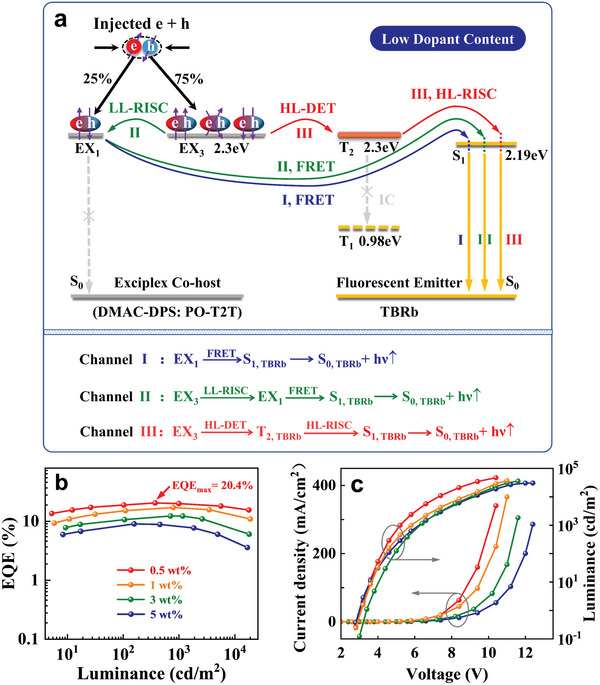
a) Schematic illustration of energy transfer mechanisms in highly efficient TBRb‐doped devices. b) EQE as a function of luminance for devices B_1_–B_4_. c) Current density–voltage–luminance (*J–V–L*) characteristics of devices B_1_–B_4_.

**Figure** **1**a presents the schematic diagram of energy transfer processes from the exciplex co‐host (DMAC‐DPS: PO‐T2T) to the TBRb emitter in highly efficient TBRb‐doped FOLEDs. The detailed structures of doped devices B_1_–B_4_ are depicted in Table S1 and Figure S1 (Supporting Information), respectively. As shown in Figure 1a, the injected holes and electrons recombine with each other due to their Coulomb attractions and subsequently reside on the neighboring host molecules of DMAC‐DPS and PO‐T2T to form the intermolecular exciplex states (EX states). As well reported in relevant literatures, the EX states include spin‐singlet exciplex (EX_1_, 25%) and spin‐triplet exciplex (EX_3_, 75%) that are almost degenerate in energy.^[^ ^10^ ^]^ Consequently, the low‐level intersystem crossing (LL‐ISC, EX_1_ → EX_3_) and LL‐RISC (EX_3_ → EX_1_) processes induced by *hyperfine* interaction can readily occur between these weakly bound EX_1_ and EX_3_ states.^[^ ^11^ ^]^ In general, the LL‐RISC process from EX_3_ to EX_1_ states dominates over these two evolutionary processes of excited states due to the much longer lifetime and a larger amount of EX_3_ than EX_1_. When the TBRb guest molecules are doped into this exciplex system, the EX states cannot directly deactivate to the ground state (S_0_) because of the concurrence of three highly efficient fast energy transfer processes from these exciplex co‐host to the TBRb guest. This would cause their energy transfer of EX_1, co‐host,_ and EX_3, co‐host_ states to the S_1, TBRb,_ and T_2, TBRb_ states of TBRb, respectively. Specifically, as the blue arrow shows in Figure 1a, the EX_1, co‐host_ state energy is transferred to the S_1, TBRb_ state through a fast Förster resonance energy transfer process (FRET, EX_1, co‐host_ → S_1, TBRb_), and then the S_1, TBRb_ state radiative transition to S_0_ generates fluorescence (channel I). Additionally, some of the EX_1, co‐host_ states formed from the EX_3, co‐host_ states through the LL‐RISC process also undergo the above process. For the sake of simple discussion, we define EX_3, co‐host_ $\overset{\;\mathrm{LL}-\mathrm{RISC}\;}{\to }$ EX_1, co‐host_ $\;\overset{\;\mathrm{FRET}\;}{\to }$ S_1, TBRb_ → S_0, TBRb_ + hν↑ as channel II, as illustrated by the green arrows. Clearly, the energy transfer of channel II is exceedingly significant for harvesting the triplet energy of exciplex co‐host to achieve efficient fluorescence emission of TBRb guest. Finally, based on the prerequisites of the close triplet energy levels between the EX_3, co‐host_ state and the T_2, TBRb_ state, the rest of EX_3, co‐host_ states can be transferred into T_2, TBRb_ by an efficient nearly‐resonant HL‐DET process, which is represented by red arrows in Figure 1a.^[^ ^12^ ^]^ The EX_3, co‐host_ state energy [*E*(EX_3, co‐host_) = 2.3 eV], the T_2, TBRb_ state energy [*E*(T_2, TBRb_) = 2.3 eV], and the T_1, TBRb_ state energy [*E*(T_1, TBRb_) = 0.98 eV]^[^ ^9^ ^]^ have been displayed in Figure 1a. Thus, $\Delta \;E_{{\mathrm{EX}}_{3,\;\mathrm{co}-\mathrm{host}}T_{2,\;\mathrm{TBRb}}}=$ 0 eV; $\Delta \;E_{{\mathrm{EX}}_{3,\;\mathrm{co}-\mathrm{host}}T_{1,\;\mathrm{TBRb}}}$= 1.32 eV. This indicates that the HL‐DET (EX_3, co‐host_ → T_2, TBRb_) process can occur effectively while the low‐level DET (EX_3, co‐host_ → T_1, TBRb_) process is negligible because the small energy difference of triplet states between host and guest is conducive to a DET process.^[^ ^13^ ^]^ Moreover, $\Delta E_{T_{2,\;\mathrm{TBRb}}T_{1,\;\mathrm{TBRb}}}$> 1 eV, and this makes the IC process from T_2, TBRb_ to T_1, TBRb_ states be heavily inhibited.^[^ ^14^ ^]^ Therefore, the T_2, TBRb_ states will be generated efficiently with no energy losses under the conditions of the strong HL‐DET process and the negligible low‐level DET and IC processes. Furthermore, due to the small energy difference $\Delta E_{S_{1,\;\mathrm{TBRb}}T_{2,\;\mathrm{TBRb}}}$ (− 0.11ev),^[^ ^9^ ^]^ the generated T_2, TBRb_ will be favorably converted into S_1, TBRb_ via the HL‐RISC process followed by the deactivation process to S_0_ for producing fluorescence (i.e., channel III). The energy transfer channel III of EX_3, co‐host_ $\overset{\;\mathrm{HL}-\mathrm{DET}\;}{\to }$ T_2, TBRb_ $\overset{\;\mathrm{HL}-\mathrm{RISC}\;}{\to }$ S_1, TBRb_→ S_0, TBRb_ + hν↑ is another important factor to achieve the high‐efficiency TBRb‐doped FOLEDs. These three channels are co‐existing but channels II and III are comparable and both are stronger than channel I, which is qualitatively discussed in Text S1 (Supporting Information). The simultaneous utilization of these three efficient energy transfer channels combining the LL‐RISC process in the exciplex co‐host with the HL‐RISC process of hot excitons in the TBRb emitter provides a powerful guarantee for achieving the high‐performance FOLEDs with the high EQE over 20%:

(1) $$\mathrm{Channel}\;I:{\mathrm{EX}}_{1,\;\mathrm{co}-\mathrm{host}}\overset{\;\mathrm{FRET}\;}{\to }\;S_{1,\mathrm{TBRb}}\to S_{0,\;\mathrm{TBRb}}+h\nu ↑$$

(2) $$\begin{array}{ccc} \mathrm{Channel}\;\mathrm{II} & : & {\mathrm{EX}}_{3,\;\mathrm{co}-\mathrm{host}}\overset{\;\mathrm{LL}-\mathrm{RISC}\;}{\to }{\mathrm{EX}}_{1,\;\mathrm{co}-\mathrm{host}}\overset{\;\mathrm{FRET}\;}{\to }S_{1,\;\mathrm{TBRb}}\to S_{0,\;\mathrm{TBRb}}\\ & & +h\nu ↑ \end{array}$$

(3) $$\begin{array}{ccc} \mathrm{Channel}\;\mathrm{III} & : & {\mathrm{EX}}_{3,\;\mathrm{co}-\mathrm{host}}\overset{\;\mathrm{HL}-\mathrm{DET}\;}{\to }T_{2,\;\mathrm{TBRb}}\overset{\;\mathrm{HL}-\mathrm{RISC}\;}{\to }S_{1,\;\mathrm{TBRb}}\to S_{0,\;\mathrm{TBRb}}\\ & & +h\nu ↑ \end{array}$$

Figure 1b displays the EQE−luminance (EQE−*L*) characteristics of the highly efficient TBRb‐doped devices B_1_–B_4_ with the EMLs of DMAC‐DPS: PO‐T2T (1: 5): x wt.% TBRb (*x* = 0.5, 1, 3, and 5), and their key performance parameters are listed in **Table** **1**. As can be seen, a maximum EQE of 20.4% is obtained in device B_1_, and other EQEs of 20.3% at 1000 cd m^−2^ and 18.5% at 5000 cd m^−2^ are also respectively acquired. Obviously, the efficiency roll‐off phenomenon at high luminance often unavoidably that existed in traditional fluorescent devices has been greatly suppressed in our devices because the quenching processes of triplet states (EX_3, co‐host_, and T_2, TBRb_) have been effectively minimized by two highly‐efficient fast energy transfer channels II and III. As far as we know, the EL efficiency of device B_1_ is one of the highest EQEs among FOLEDs with TBRb as an end emitter. However, the EQE values of the TBRb‐doped devices are significantly impaired with increasing TBRb dopant concentrations. This is attributed to the concentration quenching effect and triplet‐triplet annihilation (TTA) process at high triplet exciton density.^[^ ^15^ ^]^ Here, the triplet state refers to the high‐lying triplet (T_2, TBRb_) state of TBRb, i.e., T_2_T_2_A occurs at high TBRb dopant contents. The physical origin of T_2_T_2_A in these TBRb‐doped devices will be explored by using the fingerprint MEL probing tool. The specific discussion will be provided in **Section** **2.4**. In addition, the current density–voltage–luminance (*J*‐V–*L*) characteristics of the devices B_1_–B_4_ are given in Figure 1c, where all devices possess a relatively low turn‐on voltage of 2.9‐3.4 V at 1 cd m^−2^. The detailed parameters including normalized EL spectra and EL performances of the high‐efficiency devices B_1_–B_4_ have been presented in Figure S2 (Supporting Information) and Table 1. In the following, by characterizing the photophysical properties of solid‐state films of related materials and studying the MEL response traces from all exciplex‐based and TBRb‐doped FOLEDs, we will verify the existence of these three microscopic evolutionary channels in the highly efficient TBRb‐doped devices B_1_–B_4_.

**Table 1:** Summary of key EL performance parameters for the highly efficient devices B_1_–B_4_

Device	EML Structure	*V*_on_ ^a)^[V]	EL^b)^[nm]	EQE / CE / PE ^c)^[% / cd A^−1^/lm W^−1^]		
				Maximum	@ 1000 cd m^−2^	@ 5000 cd m^−2^
B_1_	DMAC‐DPS: PO‐T2T: 0.5 wt.% TBRb (1:5)	2.9	559	20.4 / 71.8 /44.6	20.3 / 70.4 / 42.6	18.5 / 62.0 / 30.5
B_2_	DMAC‐DPS: PO‐T2T: 1 wt.% TBRb (1:5)	2.9	560	17.2 / 60.6 / 36.6	17.0 / 60.1 / 29.6	15.1 / 52.5 / 20.1
B_3_	DMAC‐DPS: PO‐T2T: 3 wt.% TBRb (1:5)	3	564	12.3 / 43.0 / 23.0	12.1 / 42.8 / 20.4	10.6 / 37.1 / 14.2
B_4_	DMAC‐DPS: PO‐T2T: 5 wt.% TBRb (1:5)	3.1	567	9.0 / 30.3 /22.0	8.4 / 28.0 /15.1	6.4 / 21.5 / 8.9

^a)^ Turn‐on voltage at a luminance of 1 cd m^−2^

^b)^ EL emission wavelength at maximum intensity

^c)^ EQE (external quantum efficiency); CE (current efficiency); PE (power efficiency).

### 2.2 Photophysical Properties of Relevant Organic Materials in Solid‐State Films

**Figure 2:**
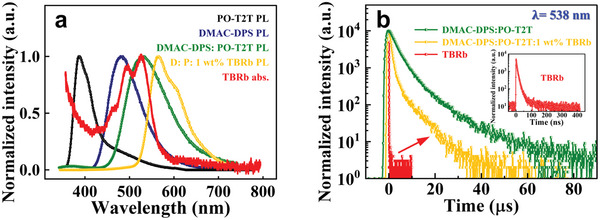
a) Absorption spectrum of TBRb and the normalized PL spectra of the DMAC‐DPS pure film, PO‐T2T pure film, DMAC‐DPS: PO‐T2T (D: P, 1: 5) blending film, and D: P: 1 wt.% TBRb doping film. b) The TPL decay curves of D: P blending film, TBRb doping film, and TBRb pure film at the detection wavelength of 538 nm. Inset: TPL curve of TBRb pure film probed within the short time range of 0–400 ns.

**Figure** **2**a presents the normalized PL spectra of PO‐T2T and DMAC‐DPS pure films, their blending film in a 1: 5 weight ratio (DMAC‐DPS: PO‐T2T, D: P = 1: 5), and three‐component TBRb doping film (D: P: 1 wt.% TBRb), as well as the absorption spectrum of TBRb. The PL emission peaks of PO‐T2T and DMAC‐DPS pure films are located at the wavelength of 387 nm and 485 nm, respectively. However, as compared to the PL spectra of PO‐T2T and DMAC‐DPS pure films, the D: P blending film has a broad and redshifted PL spectrum with the emission peak at 538 nm, which illustrates the formation of exciplex states (including singlet EX_1_ and triplet EX_3_) between the DMAC‐DPS and PO‐T2T molecules. Furthermore, the normalized PL spectrum of the D: P exciplex overlaps well with the absorption spectrum of TBRb, suggesting that the FRET process from EX_1, co‐host_ to S_1, TBRb_ states could be facilitated when the TBRb molecule is dispersed into this exciplex co‐host system.^[^ ^16^ ^]^ Actually, in the normalized PL spectrum of TBRb doping film, only the emission of TBRb with a peak at 568 nm is observed, which visually reflects the effective occurrence of this FRET process. In addition, the TPL decay curves of D: P blending film, TBRb doping film, and TBRb pure film detected at 538 nm within the range of 0–90 µs are presented in Figure 2b. The TPL decay of the D: P blending film (green curve) has a long delay lifetime at the microsecond level. It has been widely demonstrated that a long lifetime originates from the emission of EX_1_ states formed from the nonradiative EX_3_ states through the LL‐RISC process.^[^ ^10a^ ^]^ As well demonstrated in the literature, for a host‐guest system the overall TPL behavior is determined by the longer lifetime of the host or guest when there exists an energy transfer process from host to guest.^[^ ^17^ ^]^ In order to clarify the main determining factor for the transient behavior of this host‐guest system, we measured the TPL decay curves of TBRb pure film probed at 538 nm within the delay time range of 0–10 µs (red curve) and 0–0.4 µs (in the inset). As illustrated in the inset, the lifetime of the TBRb excitons is very short (≈ 20 ns). Hence, the long‐lived delay components of TBRb‐doping film (yellow curve) within the 0–90 µs time range are mainly determined by the exciplex co‐host instead of the TBRb dopant. More importantly, the faster TPL decay behavior of the TBRb doping film (yellow curve) as compared to that of the D: P blending film (green curve) identifies again the existence of an effective FRET process from EX_1, co‐host_ to S_1, TBRb_ states. Here, by studying the photophysical properties of different solid‐state films, it has been confirmed the LL‐RISC process between the EX states in the D: P exciplex co‐host and the FRET process occurred from the exciplex co‐host to the TBRb guest. That is, the evolutionary channels I and II do exist in highly efficient TBRb‐based devices. However, unfortunately, the DET process between host and guest triplet states is inoperative under optical excitation.^[^ ^18^ ^]^ In other words, in this TBRb‐doped system, the HL‐DET process in channel III that further harvests the EX_3, co‐host_ state energy to enhance the triplet energy utilization ratio cannot be observed in PL or TPL. Thus, the existence of channel III needs to be further explored by the fingerprint MEL probing tool.^[^ ^8^ ^]^ In the following, we will analyze the MEL response traces of the exciplex‐based devices A_1_–A_7_ to study their physical evolution processes.

### 2.3 Current‐Dependent MEL Traces in Exciplex‐Based Devices and Analysis of Their Physical Microscopic Processes

**Figure 3:**
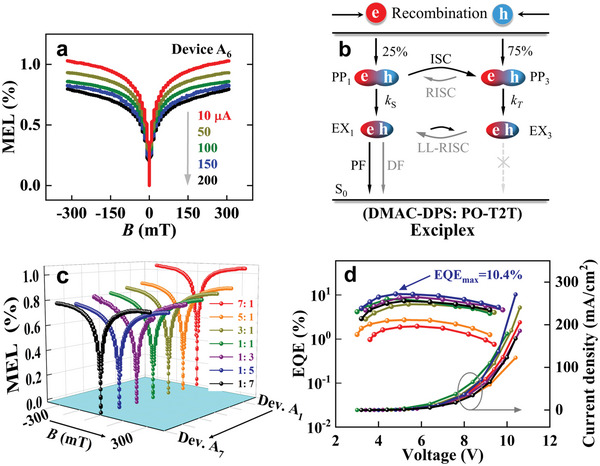
a) Current‐dependent MEL traces of device A_6_ under the current range of 10–200 µA at room temperature. b) Schematic diagram of the evolution processes of the spin‐pair states in these exciplex devices. c) The MEL traces of devices A_1_–A_7_ with various donor/acceptor ratios at 100 µA bias. d) *EQE‐V‐J* characteristic curves of devices A_1_–A_7_. PF: prompt fluorescence, DF: delayed fluorescence.

As a non‐contact and non‐destructive detection tool, MEL was widely employed to probe the physical microscopic processes that occurred in OLEDs.^[^ ^19^ ^]^ It has been well reported in the literature that the different spin‐pair state evolution processes, including ISC, RISC, TTA, and singlet fission (SF), will present various characteristic MEL response line‐shapes under an applied magnetic field *B*.^[^ ^11^, ^20^ ^]^ The commonly‐reported fingerprint MEL traces have been shown in Figure S3 (Supporting Information) when these microscopic processes exist independently in OLEDs. The current‐dependent MEL traces of the exciplex‐based device A_6_ operated at 300 K are plotted in **Figure** **3**a. As can be seen, the MEL traces of device A_6_ always exhibit a *B*‐mediated ISC line‐shape within the bias current range of 10–200 µA. Specifically, the MEL traces show a sharp increase near zero magnetic field, and then approach saturation with the further enhancement of the external *B*. This indicates that the ISC process is dominated in device A_1_. Furthermore, these MEL amplitudes gradually reduce with increasing the bias currents, which is a so‐called normal current dependence in the literature.^[^ ^21^ ^]^ A similar phenomenon is also separately presented in devices A_1_‐A_5_, and A_7_, as summarized in Figure S4 (Supporting Information).

To reveal the physical origin of the ISC‐dominated MEL response curves from exciplex‐based devices A_1_–A_7_, a schematic diagram for the spin‐pair state evolution processes in these exciplex devices is exhibited in Figure 3b. The injected holes (*e*) and electrons (*h*) first form intermolecular polaron‐pair states (PP states) between DMAC‐DPS and PO‐T2T molecules under their Coulomb attractions, which consist of the energy‐degenerated singlet (PP_1_) and triplet (PP_3_) states.^[^ ^22^ ^]^ Due to the coupling of the spins of *e* and *h* polarons with the spins of hydrogen nuclei in the organic host molecules, a locally and randomly oriented *hyperfine* field is generated. The spins of *e* or *h* polarons process around the *hyperfine* field, leading to spin mixing processes (i.e., ISC and RISC) of *e‐h* pairs with different spin quantum numbers (S = 0 for PP_1_ and S = 1 for PP_3_ states). In organic semiconductor molecules, the *hyperfine* field is typically several milli‐Tesla. Meanwhile, PP_1_ and PP_3_ states will further form weakly bound EX_1_ and EX_3_ states at unequal rate constants (*k*_S_ and *k*_T_, *k*_S_ < *k*_T_), respectively.^[^ ^23^ ^]^ Consequently, the ISC process dominates the spin mixing processes between the PP states due to the inequality of *k*_S_ < *k*_T_, although both ISC and RISC processes occur simultaneously. In addition, as discussed in Section 2.2, the evolutionary processes between the EX states are dominated by the LL‐RISC process because EX_3_ state has a longer lifetime than EX_1_ state. Finally, the EX_1_ states formed directly from PP_1_ states or up‐converted from the EX_3_ states by the LL‐RISC process will be deactivated into S_0_ state to emit fluorescence (PF and DF). For MEL measure results of exciplex‐based devices where the rate of ISC process between the PP states is higher than the rate of LL‐RISC process between the EX states, the total MEL will exhibit an ISC‐dominated response curve because the MEL response is an overlapping effect between the *B*‐mediated polaron ISC and exciplex RISC channels in devices A_1_–A_7_ and ISC and RISC processes have opposite MEL line‐shapes.

According to the literature report, in an exciplex‐based device, balanced carrier injection can greatly suppress the quenching of EX_3_ states by free carriers and promote the LL‐RISC process from EX_3_ to EX_1_.^[^ ^11^ ^]^ In other words, under the same operating bias‐current, exciplex‐based devices with balanced carrier injection will show a comparatively weaker positive strength of the MEL trace. For comparing the MEL strength from different exciplex devices, the MEL traces of devices A_1_–A_7_ operating at 100 µA are displayed in Figure 3c, and the specific MEL response values obtained at *B* = 9 mT are shown in Figure S5 (Supporting Information). The MEL magnitudes for devices A_1_–A_7_ acquired at 9 mT are a more accurate reflection as to the intensity of the ISC process occurring within the devices. This is because the characteristic linewidth of the MEL curves for these spin mixing processes (ISC and RISC) is usually several milli‐Tesla. As shown in Figure 3c, with the weight ratio of DMAC‐DPS: PO‐T2T changes from 7:1 to 1:7, the MEL curve of the corresponding exciplex‐based device first shows a decreasing and then an increasing ISC line‐shape. Among devices A_1_–A_7_, the smallest MEL amplitude is observed in device A_6_ (Figure S5, Supporting Information), indicating that the most balanced carrier injection happens in this device. This will also be reflected in its photoelectric performance. The EQE‐*V‐J* characteristics curves of the exciplex devices A_1_–A_7_ are presented in Figure 3d. As a supplementary, the EL spectra and key EL parameters of devices A_1_–A_7_ are summarized in Figure S6 and Table S2 (Supporting Information), respectively. As can be seen from Figure 3d, with increasing the proportion of the PO‐T2T component in the EML, the maximum EQE value of the corresponding device increases and then decreases gradually. This is because the excessive DMAC‐DPS or PO‐T2T components will cause the hole‐rich or electron‐rich EML of the device, respectively, both of which will aggravate the quenching of the EX_3_ states by free carriers and thus impair device performance.^[^ ^24^ ^]^ Obviously, device A_6_ [DMAC‐DPS: PO‐T2T (1: 5)] exhibits a relatively high EQE_max_ value of 10.4% and exceeds five times that of device A_1_. Comparing the EL performances of devices A_1_–A_7_ in Table S2 (Supporting Information), it can be found that device A_6_ has the optimal EL performance because the balanced carrier injection improves the utilization ratio of the EX_3_ states via suppressing the quench of EX_3_ states by excessive charges.^[^ ^25^ ^]^ This is the reason why we dope TBRb molecules into the D: P (1: 5) mixing matrix as an EML to fabricate highly‐efficient TBRb‐doped FOLEDs. After we analyze the underlying mechanism for the ISC‐dominated MEL response traces from exciplex‐based devices A_1_–A_7_, Next, the physical evolution process in the TBRb‐doped devices B_1_–B_4_ will be further investigated using fingerprint MEL probing tools.

### 2.4 Analyses of Current‐, Concentration‐, and Temperature‐Dependent MEL Traces in TBRb‐Based Devices B_1_–B_4_

**Figure 4:**
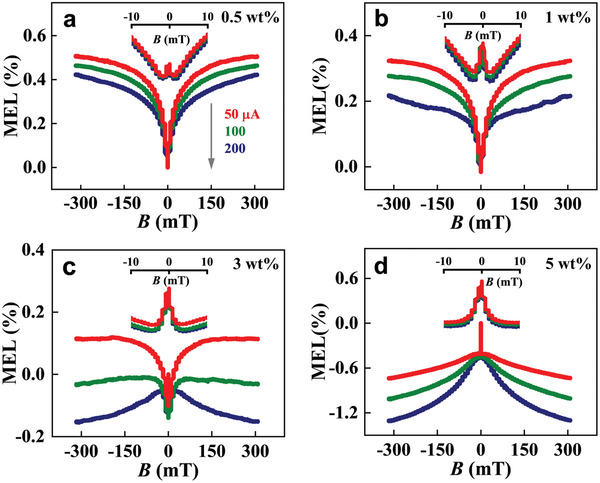
a–d) Current‐dependent MEL traces of TBRb‐doped devices B_1_–B_4_ with different TBRb dopant contents at 300 K.

**Figure 5:**
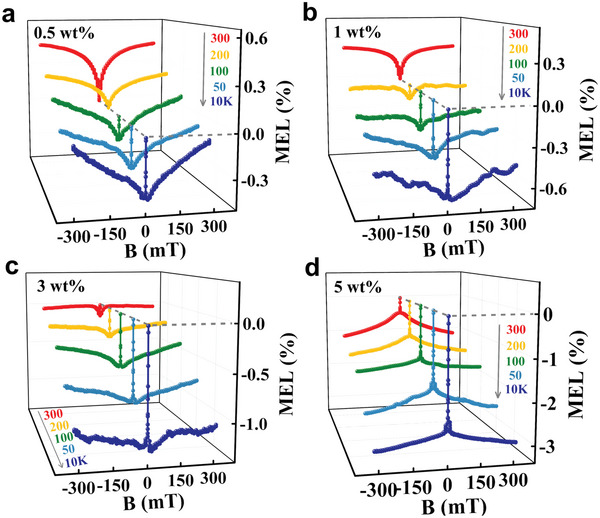
a–d) Temperature‐dependent MEL traces of TBRb‐doped devices B_1_–B_4_ at a bias‐current of 100 µA.

The current‐dependent MEL traces from TBRb‐doped devices B_1_–B_4_ with different TBRb dopant concentrations (0.5, 1, 3, and 5 wt.%) are depicted in **Figure** **4**, and their detailed MEL structures within the *B* range from −9 to 9 mT are displayed in the insets of Figure 4, respectively. According to the difference in MEL line‐shapes within various *B* ranges, these MEL traces can be divided into low field effect (MEL_L_, |*B*| ≤ 9 mT) and high field effect (MEL_H,_ 9 < |*B*| ≤ 300 mT) so that they will be analyzed separately.^[^ ^8^ ^]^ First, we will investigate the current‐dependent MEL_L_ traces from devices B_1_–B_4_. As can be seen in the insets of Figure 4a–c, all MEL_L_ curves consist of a superposition of a narrow Lorentzian line‐shapes with 4 mT FWHM (full width at half maximum) and a wide inverted Lorentzian one with an FWHM of 9 mT. Moreover, their amplitudes of narrow Lorentzian line‐shapes enhance with the increase of TBRb dopant content. Specifically, as |*B*| increases from 0 to 9 mT, all MEL_L_ response curves of devices B_1_‐B_3_ exhibit a sharp decrease in the range from 0 to 4 mT and reach a minimum at 4 mT, followed by a monotonic increase from 4 to 9 mT. Furthermore, the MEL_L_ traces of device B_4_ show a sharp decrease from 0 to 4 mT but present no change in the range of 4–9 mT. This is a typical RISC‐dominated MEL line‐shape as shown in the inset of Figure 4d. As a comparison, the MEL_L_ components of the non‐doped device A_6_ within the *B* range from −9 to 9 mT are plotted in Figure S7 (Supporting Information). Each curve presents an inverted Lorentzian line‐shape with an FWHM of 9 mT, which is an ISC‐dominated MEL trace. In brief, along with the increase of TBRb dopant concentrations from 0 to 5 wt.%, the MEL_L_ characteristics of the corresponding devices gradually turn from a positive ISC line‐shape to a negative RISC one. It is worth noticing that the only difference in the structures of devices B_1_–B_4_ and device A_6_ exists in their EMLs, that is, devices B_1_–B_4_ are doped with TBRb dopant but device A_6_ is only based on the exciplex co‐host (i.e., without any TBRb dopant molecules in its EML). Accordingly, it is reasonable for us to propose that a strong RISC process occurs in TBRb dopant molecules and this process contributes to the rich MEL_L_ structures of doped devices B_1_–B_4_. This is because the MEL trace is the superposition result of *B*‐mediated evolutionary processes involved in both exciplex co‐host and TBRb guest, and the superposition schematic diagram is shown in Figure S8 (Supporting Information). To quantify the contribution of the MEL response generated from TBRb molecules to the MEL traces of devices B_1_–B_4_, we subtracted the MEL response data of the un‐doped device A_6_ from the MEL response data of TBRb‐doped devices B_1_–B_4_, and their resultant MEL curves are depicted in Figure S9 (Supporting Information). Obviously, all MEL_L_ curves show a RISC‐like line‐shape, and their response magnitudes increase with the proportion of TBRb dopants, indicating that the RISC process observed in the MEL curves of TBRb‐doped devices B_1_–B_4_ is indeed originated from TBRb molecules. However, the lowest singlet‐triplet energy splitting of TBRb molecules ($\Delta E_{S_{1}T_{1}}$ = 1.21 eV) is extremely large, which forbids the conversion process from T_1, TBRb_ states to S_1, TBRb_ states under electrical excitation conditions. Therefore, the RISC process generated in TBRb molecules can stem only from the conversion from T_2, TBRb_ to S_1, TBRb_ (namely HL‐RISC process, T_2, TBRb_ → S_1, TBRb_). In addition, the monotonic enhancement in the negative MEL_L_ strength of TBRb‐doped devices B_1_–B_4_ at low temperatures also verifies this HL‐RISC process from T_2, TBRb_ to S_1, TBRb_, as shown in **Figure** **5**. As a comparison, the temperature‐dependent MEL curves of non‐doped device A_6_ are given in Figure S10 (Supporting Information). It has been reported in the literature that the RISC rate constant *k*_RISC_ can be expressed as follows^[^ ^10a^ ^]^:

(4) $$k_{\mathrm{RISC}}\propto \exp\left(-\Delta E_{\mathrm{ST}}/k_{B}T\right)$$

where Δ*E*_ST_, *k*_B_, and T are the singlet‐triplet energy difference, Boltzmann's constant, and temperature, respectively. Due to the energy difference $\Delta \;E_{S_{1}T_{2}}$= −0.11 eV < 0 eV in TBRb, *k*_HL‐RISC_ will increase with reducing operational temperatures due to their prolonged lifetime of T_2, TBRb_. That is, the rate of the exothermic HL‐RISC process will get improved at low temperatures, which is in consonance with the temperature‐dependent MEL traces of the TBRb‐doped devices B_1_–B_4_ (**Figure** **5**). Furthermore, we contrastingly doped the thermally‐activated delayed fluorescence material 2,3,5,6‐tetrakis(3,6‐diphenylcarbazol‐9‐yl)−1,4‐dicyanobenzene (4CzTPN‐Ph) with 5 wt.% content into the DMAC‐DPS: PO‐T2T co‐host for eliminating the RISC signal originated from the LL‐RISC process (EX_1_←EX_3_), as shown in Figure S11 (Supporting Information). In this case, similar to the TBRb‐doped devices, the FRET process from EX_1_ to S_1, 4CzTPN‐Ph_ can occur efficiently. As can be seen, the MEL traces of the 4CzTPN‐Ph doped device show no RISC signals (Figure S11b, Supporting Information). This is because 4CzTPN‐Ph does not have the HL‐RISC evolution process. Based on the above two experimental facts, we believe that the RISC signal of the MEL traces observed in the TBRb‐doped devices B_1_–B_4_ comes from the HL‐RISC from T_2, TBRb_ to S_1, TBRb_. More importantly, the existence of the HL‐RISC process provides direct evidence for the occurrence of the HL‐DET process, since the T_2, TBRb_ state is generated by the HL‐DET process that harvests the EX_3, co‐host_ state energy.^[^ ^26^ ^]^ Therefore, the existence of effective energy transfer channel III (EX_3, co‐host_ $\overset{\;\mathrm{HL}-\mathrm{DET}\;}{\to }$ T_2, TBRb_ $\overset{\;\mathrm{HL}-\mathrm{RISC}\;}{\to }$ S_1, TBRb_ → S_0, TBRb_ + hν↑) in the highly efficient TBRb‐doped devices B_1_–B_4_ was successfully verified by the MEL visualization tool. The utilization of channel III is a method to further harness the non‐radiative EX_3, co‐host_ state energy, and it is also the key channel for the EQE value of the high‐efficiency device B_1_ to be above 20%.

The specific analysis for the current‐dependent MEL_H_ traces from devices B_1_–B_4_ is discussed as follows. As shown in **Figure** **4**, the MEL_H_ from devices B_1_‐B_2_ with low TBRb dopant contents reveals a fast saturation along with the enhancement of *B* value under different bias currents. In contrast, the MEL_H_ of devices B_3_‐B_4_ with higher dopant contents show a gradual decrease at high bias currents (such as 200 µA). This is consistent with the characteristic MEL_H_ line‐shape of the TTA process reported in the literature (as displayed in Figure S3, Supporting Information).^[^ ^15^ ^]^ The MEL_H_ traces with *B*‐mediated TTA line‐shape suggest that a TTA process occurs in devices B_3_‐B_4_. Some research work reported that the TTA process was originated from T_1_ exciton of the emitter.^[^ ^27^ ^]^ However, in our TBRb‐doped system, this TTA signal comes from the T_2_T_2_A process of TBRb (see Text S2, Supporting Information, for details). We propose that as the fraction of TBRb dopants increases, it is unavoidable that the enhanced HL‐DET process leads to an increscent T_2, TBRb_ exciton density. Moreover, the shorter intermolecular spacing distance of TBRb guest molecules promotes the high‐density T_2, TBRb_ excitons to efficiently annihilate through the T_2_T_2_A process (T_2, TBRb_+ T_2, TBRb_ $\overset{{\;T}_{2}T_{2}A\;}{\to }$ S_n, TBRb_, Figure S12, Supporting Information). Subsequently, the S_n, TBRb_ state rapidly relaxes to the lowest singlet state S_1, TBRb_ via the IC channel, and then deactivates to S_0_ state via emitting fluorescence.^[^ ^28^ ^]^ Compared to the HL‐RISC process, the existence of the T_2_T_2_A process undoubtedly causes a reduction in T_2, TBRb_ exciton utilization ratio, and thus impairs the device efficiency. Additionally, we guess the aggregation of TBRb molecules at relatively large doping concentrations will lead to a trivial decrease in the EL efficiency of the device. To verify this conjecture, we measured the PL quantum yields (PLQYs) of TBRb‐doped films with different doping concentrations, as shown in Figure S13 (Supporting Information). As can be seen, the PLQYs of the corresponding films decrease slightly with increasing doping concentration. The weak reduction of PLQY is attributed to the concentration quenching effect.^[^ ^15b^ ^]^ More details are provided in Text S3 (Supporting Information). Taken together, the strong triplet annihilation and weak concentration quenching effects jointly lead to the decline in dopant‐concentration‐dependent EQEs from TBRb‐doped devices. Obviously, in devices B_1_‐B_2_ with low TBRb dopant concentrations, only the HL‐RISC processes occur on the TBRb molecules. However, in devices B_3_‐B_4_ with high doping concentrations, the HL‐RISC, T_2_T_2_A, and concentration quenching effects occur simultaneously on the TBRb molecules. Therefore, a low TBRb dopant content is necessary to suppress the T_2, TBRb_ exciton annihilation and then achieve an improvement in device efficiency. The ceaseless generation of the S_1, TBRb_ state is guaranteed by utilizing three efficient fast energy transfer channels to fully harvest the exciplex co‐host energy. As a result, both high EQE and low‐efficiency roll‐off are simultaneously achieved in the TBRb‐based FOLEDs, as depicted in Figure 1b.

## 3 Conclusion

In this work, highly efficient and low‐efficiency roll‐off FOLEDs were obtained by doping TBRb emitters into an energy‐matched exciplex co‐host system. A maximum EQE value of 20.4% is successfully achieved in a 0.5 wt.% TBRb‐doped FOLED, which is the largest EQE ever recorded for FOLEDs without any sensitizer and made only from fully fluorescent materials with TBRb as an end emitter. Such high EQE value and low‐efficiency roll‐off are attributed to the synergistic effects of three efficient fast energy transfer channels, which fully utilize the non‐radiative triplet states energy via combining the LL‐RISC in exciplex co‐host and the HL‐RISC of hot excitons in TBRb guest. Furthermore, the existence of these three efficient energy transfer channels was successfully verified by studying the photo‐physical properties of the materials and their fingerprint MEL traces of these devices. Our work not only enriches the physics understanding of the HL‐RISC channel in TBRb, i.e., “hot exciton” channel, but also opens a new approach to designing high‐efficiency and low‐efficiency‐roll‐off FOLEDs based on the traditional yellow‐color fluorescent emitter of TBRb for lighting sources and flat panel displays.

## 4 Experimental Section

FOLEDs involved in this work have the following device architectures: indium tin oxide (ITO) / poly(3,4‐ethylenedioxythiophene):poly(styrene‐sulfonate) (PEDOT: PSS, 30 nm) / N,N’‐bis (naphthalen‐1‐yl)‐N,N’‐bis(phenyl)benzidine (NPB, 15 nm) / tris(4‐carbazoyl‐9‐ylphenyl)amine (TCTA, 15 nm) / DMAC‐DPS (10 nm) / EML (60 nm) /PO‐T2T (40 nm) / lithium fluoride (LiF, 1 nm) / Al (100 nm). The specific EML configurations and corresponding device names have been listed in Table S1 (Supporting Information). For device preparation, the ITO‐patterned glass substrates were first cleaned by sequential ultra‐sonication in detergent, deionized water, anhydrous ethanol, and acetone. Subsequently, PEDOT:PSS was spin‐coated onto the cleaned ITO‐patterned substrate and then annealed at 120 °C for 20 min to remove residual water. After these procedures, the sample substrates were quickly transferred to a high vacuum molecular beam deposition (MBD) system. All the organic functional layers and the composite cathode of LiF/Al were fabricated by the MBD technology at a base pressure (≈10^−6^ pa). Multi‐component mixing layers were made by co‐deposition. In addition, the solid‐state films were prepared by depositing the corresponding materials onto quartz substrates for a series of optical detection.

After device fabrication, the current density–voltage–luminance characteristics were evaluated by a Keithley 2400 source meter and a luminance colorimeter (CS‐160). The electroluminescence (EL) spectra of these FOLEDs were measured using a SpectraPro‐2300i spectroscopy. The EQEs were calculated from the corresponding EL spectra, current density, and luminance data. For the MEL measurement [MEL(*B*) = [EL(*B*) – EL(0)] / EL(0), where EL(*B*) and EL(0) are the EL intensity of the device with and without an external magnetic field, respectively, the devices were mounted on the cold finger of a closed‐cycle cryostat (Janis: CCS‐350S). The Keithley 2400 source meter and an electromagnet (powered by the Lakeshore EM647 unit) were used to provide the constant device bias and the tunable external magnetic field (*B*), respectively. The EL intensity and the corresponding *B* value were detected by a photomultiplier tube and a Hall sensor. In addition, the operating temperatures of the devices were controlled by a temperature controller (Lakeshore 331). Finally, the MEL traces were plotted in accordance with their definition. The photoluminescence (PL) spectra and the transient PL (TPL) decay of solid‐state films were measured with an Edinburgh fluorescence spectrometer (FLS1000).

## Conflict of Interest

The authors declare no conflict of interest.

## Supporting information

Supporting Information

## Data Availability

The data that support the findings of this study are available from the corresponding author upon reasonable request.
