# Supplementary material for: High‐Performance Hot‐Exciton OLEDs via Fully Harvesting Triplet Excited States from Both the Exciplex Co‐Host and the TBRb Emitter
Source: Adv Sci (Weinh). 2023 Aug 16;10(29):2303192. doi: 10.1002/advs.202303192 (PMC10582462; doi:10.1002/advs.202303192)
Supplement: Supplementary file 1 — Supporting Information [file ADVS-10-2303192-s001.pdf]

ADVANCED SCIENCE
Open Access

Supporting Information

for *Adv. Sci.*, DOI 10.1002/advs.202303192

High-Performance Hot-Exciton OLEDs via Fully Harvesting Triplet Excited States from Both the Exciplex Co-Host and the TBRb Emitter

*Fuxian Wei, Jing Chen, Xi Zhao, Yuting Wu, Huiyao Wang, Xiaoli Chen and Zuhong Xiong**

Supporting Information

High-Performance Hot-Exciton OLEDs via Fully Harvesting Triplet Excited States from Both the Exciplex Co-Host and the TBRb Emitter

*Fuxian Wei, Jing Chen, Xi Zhao, Yuting Wu, Huiyao Wang, Xiaoli Chen, and Zuhong Xiong**

Chongqing Key Laboratory of Micro&Nano Structure Optoelectronics, School of Physical Science and Technology, Southwest University, Chongqing 400715, P. R. China

* E-mails: zhxiong@swu.edu.cn

Supporting Texts

Text S1: The strength relationship of channels I-III in TBRb-doped devices.

In the cohost-guest system of this work, three channels are co-existing but channels II and III are comparable and both are stronger than channel I, qualitatively. The specific reasons are described as follows. As we can see from Figure 3d, for pure exciplex-based OLEDs (devices A_1-A_7), the maximum EQE of the exciplex co-host OLEDs is around 10%, that is, channels I and II produce 10% EQE together. Theoretically, channel I has the maximum EQE of 5% when EX_1 and EX_3 have equal formation rates because the quantity of EX_3 is three times that of EX_1 according to the spin statistic rule. Thus, we can conclude that channel I and II have 5.0% EQE, respectively, when there is no TBRb dopant in the pure exciplex-based OLEDs. When TBRb is doped into the exciplex co-host of DMAC-DPS: PO-T2T, from Figure 1b we can see that the TBRb-doped OLEDs has the maximum EQE of near 20%. Thus, the EQE enhancement due to the occurrence of channel III of the TBRb dopant is 10% if channel II (LL-RISC, $EX_1 \leftarrow EX_3$) is not changed after the TBRb dopant is doped into the exciplex co-host of DMAC-DPS: PO-T2T. In this case, channel III is the strongest one among channels I-III. However, as reported in the literature, channel II will become stronger after TBRb is doped into the exciplex co-host because EX_1 states will quickly evolve onto by the S_1 excitons via a fast FRET process occurring from the exciplex co-host to the TBRb dopant.[1] Therefore, channel III reduces because the HL-DET process decreases. As a result, channels II and III are comparable and both are stronger than channel I.

Text S2: Source of the TTA signal for the MEL curves of the devices B_3-B_4.

Some work focus on the dynamics of excitons in "hot exciton" materials when they are used as purely emitting layer or host.[1, 2] For "hot exciton" materials, the HL-RISC process from T_n to S_1 states is in competition with the IC process from T_n to T_{n-1} states. Their rate constants ($k_{HL\text{-}RISC}$ and k_{IC}) are inversely proportional to the energy difference (ΔE) between their respective initial and final states. Theoretically, the generation of T_{n-1} will be negligible when $k_{HL\text{-}RISC}$ is much larger than k_{IC}. However, this condition is difficult to meet, as reported in these articles. On one hand, the T_{n-1} (n= 2) excitons can be generated by the IC process due to the relatively large k_{IC}. On the other hand, the generation of T_1 excitons is also promoted because of the aggregation of many host matrixes in the emitting layer. In this case, the T_1 exciton density will be increased, especially at high current densities. Therefore, it is reasonable that the T_1T_1A process will occur, as demonstrated in these works. Nevertheless, in our work,

the TTA signal of the MEL curves from TBRb-doped devices originates from $T_{2,\ TBRb}$ instead of $T_{1,\ TBRb}$. Firstly, for TBRb, a very small $\Delta E_{S_1T_2}$(− 0.11ev) and a large $\Delta E_{T_2T_1}$(1.32 eV) lead to a very large $k_{HL\text{-}RISC}$ / k_{IC}. This causes almost all $T_{2,\ TBRb}$ excitons to be converted into $S_{1,\ TBRb}$ by the HL-RISC process, rather than to $T_{1,\ TBRb}$ via the IC channel. Secondly, we doped TBRb with low concentration into a host as an emitting layer, which can further suppress the IC process as demonstrated in the literature.[3] In this case, the generated $T_{1,\ TBRb}$ excitons is negligibe. Therefore, the TTA signal of the MEL curves does not come from the $T_{1,\ TBRb}$ but $T_{2,\ TBRb}$ states.

Text S3: Origin of the EQE decline for the TBRb-doped devices.

As can be seen in **Figure S13**, the PLQYs of the corresponding films decrease slightly with increasing doping concentration. We guess the weak reduction of PLQY is attributed to the concentration quenching effect. [4] That is, the aggregation of TBRb molecules at relatively large doping concentrations will lead to a trivial decrease in the EL efficiency of the device. However, this is not the major factor leading to the decline in the device EQE. The EQE of an OLED can be expressed as follows:[5] $\mathrm{EQE} = \gamma \times \eta_{PL} \times \eta_r \times \eta_{out}$, where γ is the balance factor of injected hole and electron charges (γ= 1 for balanced devices), η_{PL} is the PLQY of film materials, η_r is the efficiency of radiative exciton production, and η_{out} is the light-out-coupling efficiency (20~30%).[6] As the TBRb doping concentration increased from 0.5 to 5 wt%, the PLQY values of the corresponding films decreased by about 9%. Thus, the reduction in EQE caused by the TBRb aggregation should be theoretically less than 2.7%. However, the EQE maximum declined by about 11% from device B_1 to B_4. Therefore, there are other factors for the EQE decline of the devices under an electrical excitation. We believe that the major cause is the triplet annihilation of TBRb (T_2T_2A) by analyzing the high-field MEL components from TBRb-doped devices (Figure 4). This is because, as compared to the HL-RISC channel ($S_1 \leftarrow T_2$, i.e., one hot exciton T_2 produces one emissive singlet S_1), T_2T_2A ($T_2+T_2 \rightarrow S_1+S_0$) is the process in which two hot excitons generate one singlet S_1. Thus, in competition with the HL-RISC of hot excitons, the T_2T_2A process reduces the yield of radiative excitons leading to lower EQE values of the TBRb-doped devices. Taken together, the weak concentration quenching and strong triplet annihilation effects jointly lead to the decline in dopant-concentration-dependent EQEs from TBRb-doped devices.

Supporting Tables:

Table S1. Specific structures of exciplex-based and TBRb-doped FOLEDs involved in the main text: ITO / PEDOT: PSS / NPB (15 nm) / TCTA (15 nm) / DMAC-DPS (10 nm) / EML (60 nm) / PO-T2T (40 nm) / LiF (1 nm) / Al.

Device name	EML
A_1	DMAC-DPS: PO-T2T (7:1)
A_2	DMAC-DPS: PO-T2T (5: 1)
A_3	DMAC-DPS: PO-T2T (3: 1)
A_4	DMAC-DPS: PO-T2T (1: 1)
A_5	DMAC-DPS: PO-T2T (1: 3)
A_6	DMAC-DPS: PO-T2T (1: 5)
A_7	DMAC-DPS: PO-T2T (1: 7)
B_1	DMAC-DPS: PO-T2T: 0.5 wt% TBRb (1: 5)
B_2	DMAC-DPS: PO-T2T: 1 wt% TBRb (1: 5)
B_3	DMAC-DPS: PO-T2T: 3 wt% TBRb (1: 5)
B_4	DMAC-DPS: PO-T2T: 5 wt% TBRb (1: 5)
B_5	TBRb

Table S2. Summary of EL performances from exciplex-based devices A_1-A_7.

Device	EML Structure	V_{on} [V]	EL [nm]	EQE / CE / PE [% / cd A^{-1}/ lm W^{-1}]		
				Maximum	@ 1000 cd m^{-2}	@ 5000 cd m^{-2}
A_1	DMAC-DPS: PO-T2T (7: 1)	3.4	529	2.0 / 6.7 / 4.5	1.8 / 6.3 / 3.0	-- / -- / --
A_2	DMAC-DPS: PO-T2T (5: 1)	3.3	532	2.7 / 9.4 / 6.7	2.4 / 8.2 /3.7	-- / -- / --
A_3	DMAC-DPS: PO-T2T (3: 1)	3.2	538	7.1 / 20.1 / 12.8	7.0 / 20 / 10.9	6.2 / 18.0 / 7.6
A_4	DMAC-DPS: PO-T2T (1: 1)	3.2	540	8.1 / 20.6 / 20.4	6.3 / 18.2 / 10	6.0 / 10.2 / 5.4
A_5	DMAC-DPS: PO-T2T (1: 3)	3	536	9.0 / 26.3 / 16.4	8.6 / 25.5 / 12.7	6.5 / 19.2 / 7.7
A_6	DMAC-DPS: PO-T2T (1: 5)	2.9	538	10.4 / 32.2 / 21.2	9.9 / 30.6 / 16.6	7.2 / 21.5 / 9.0
A_7	DMAC-DPS: PO-T2T (1: 7)	3.1	530	7.6 / 24.3 / 15.9	6.8/ 23.2 / 11.8	4.4 / 15.2 / 5.7

Supporting Figures:

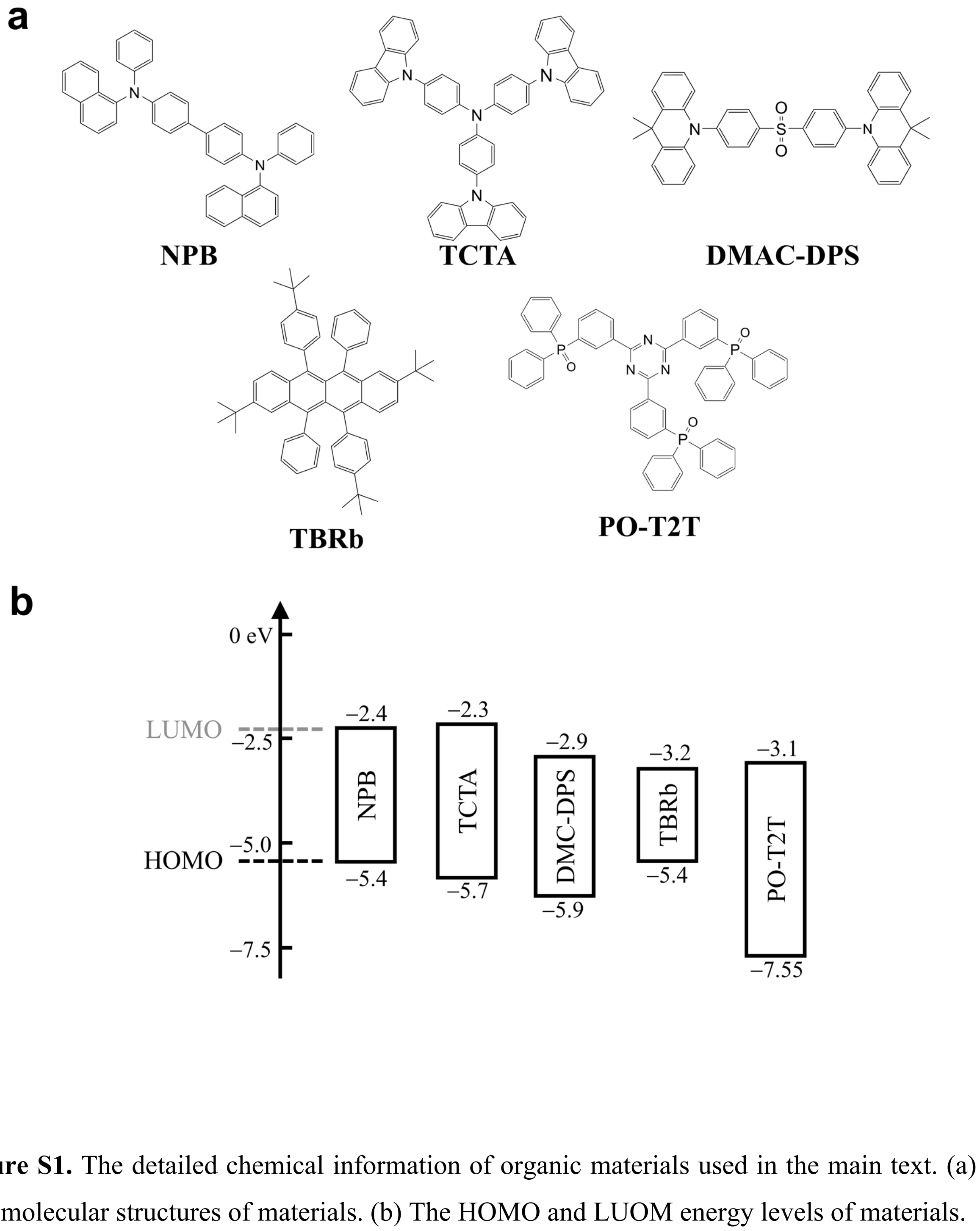

Figure S1. The detailed chemical information of organic materials used in the main text. (a) The molecular structures of materials. (b) The HOMO and LUOM energy levels of materials.

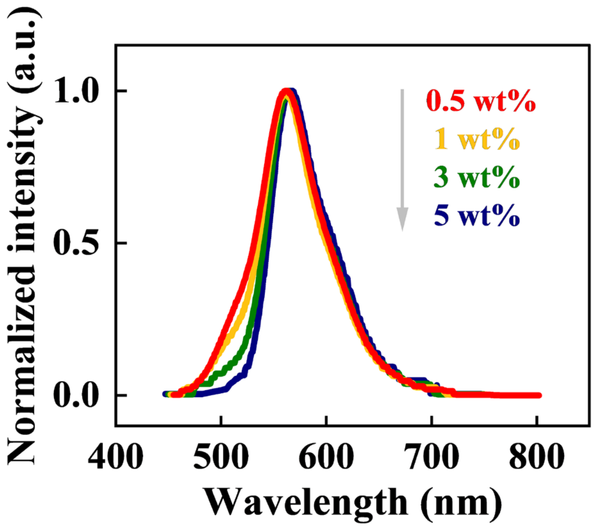

Figure S2. Normalized EL spectra of TBRb-based devices B1-B4 with different TBRb dopant contents at 300 K.

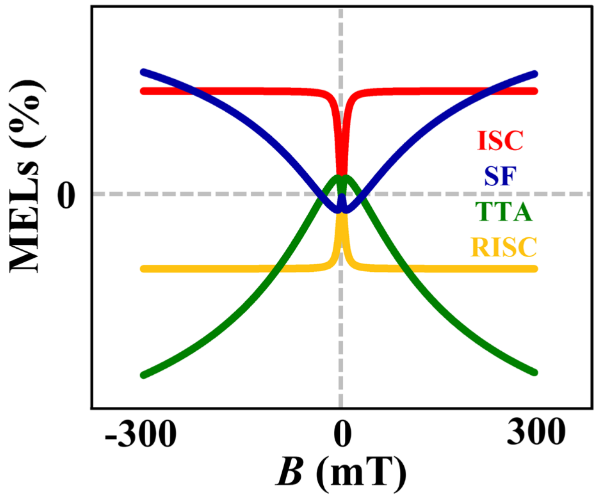

Figure S3. Schematic diagrams of fingerprint MEL line-shapes corresponding to various microscopic evolution process (including ISC, RISC, SF, and TTA process) of spin-pair states.

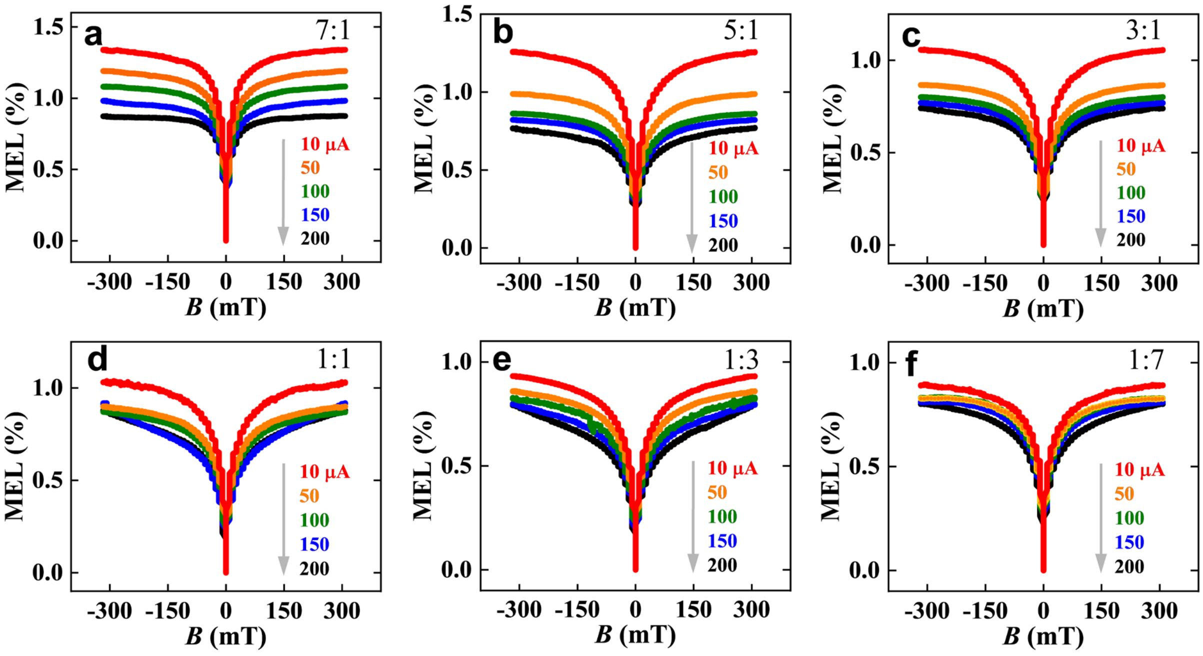

Figure S4. (a-f) Current-dependent MEL traces from exciplex-based devices A_1-A_5, and A_7 with different D: P weight ratios at 300K.

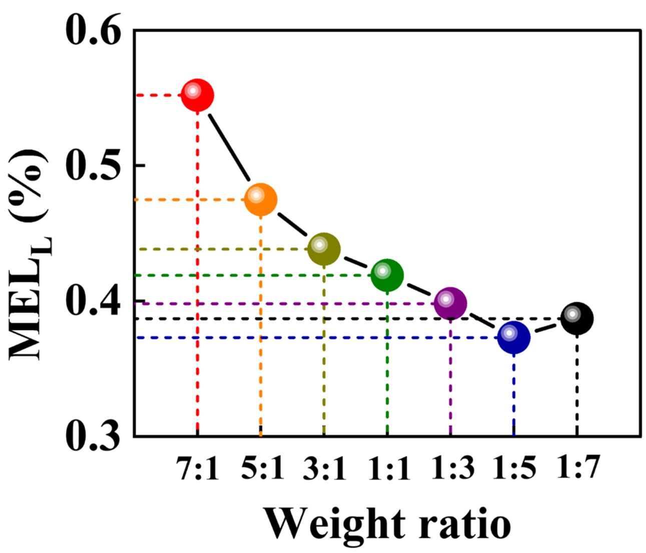

Figure S5. The MEL response values (B= 9 mT) of exciplex-based devices A_1-A_7 with different weight ratio at a bias-current of 100 μA.

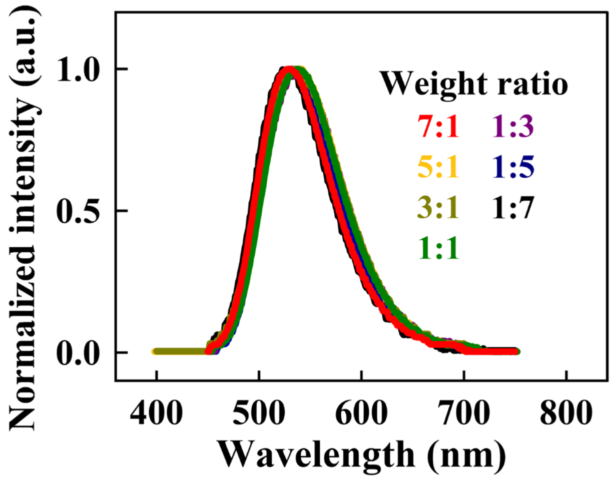

Figure S6. Normalized EL spectra of exciplex-based devices A_1-A_7.

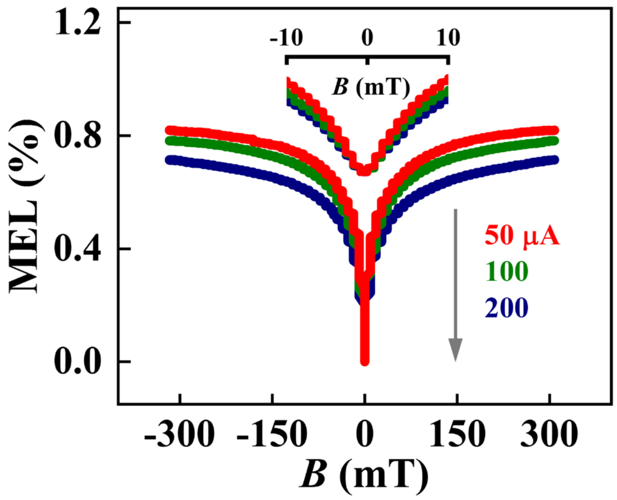

Figure S7. Room temperature current-dependent MEL traces of exciplex-based device A_6, and its inset shows the MEL details within the *B* range of − 9 mT to 9 mT.

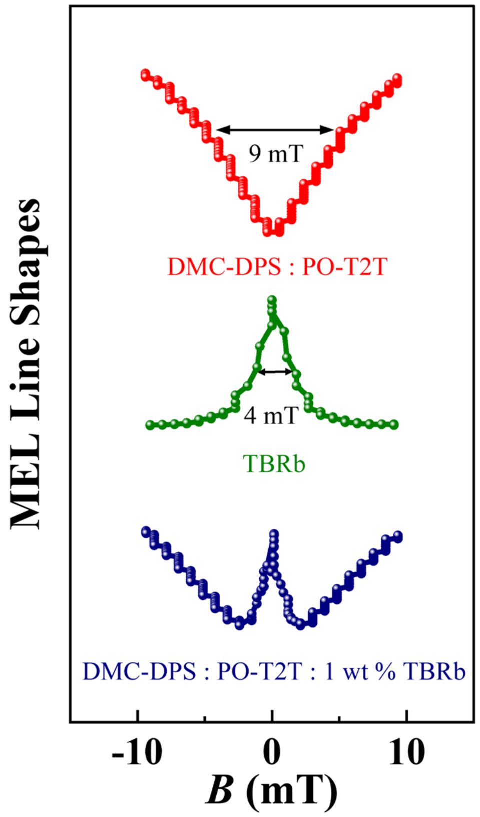

Figure S8. Superimposed diagram of ISC and RISC line shapes with different linewidths. The MEL curves in this figure are acquired from devices A_6 (red), B_5 (green) and B_2 (blue) at 100 μA, respectively.

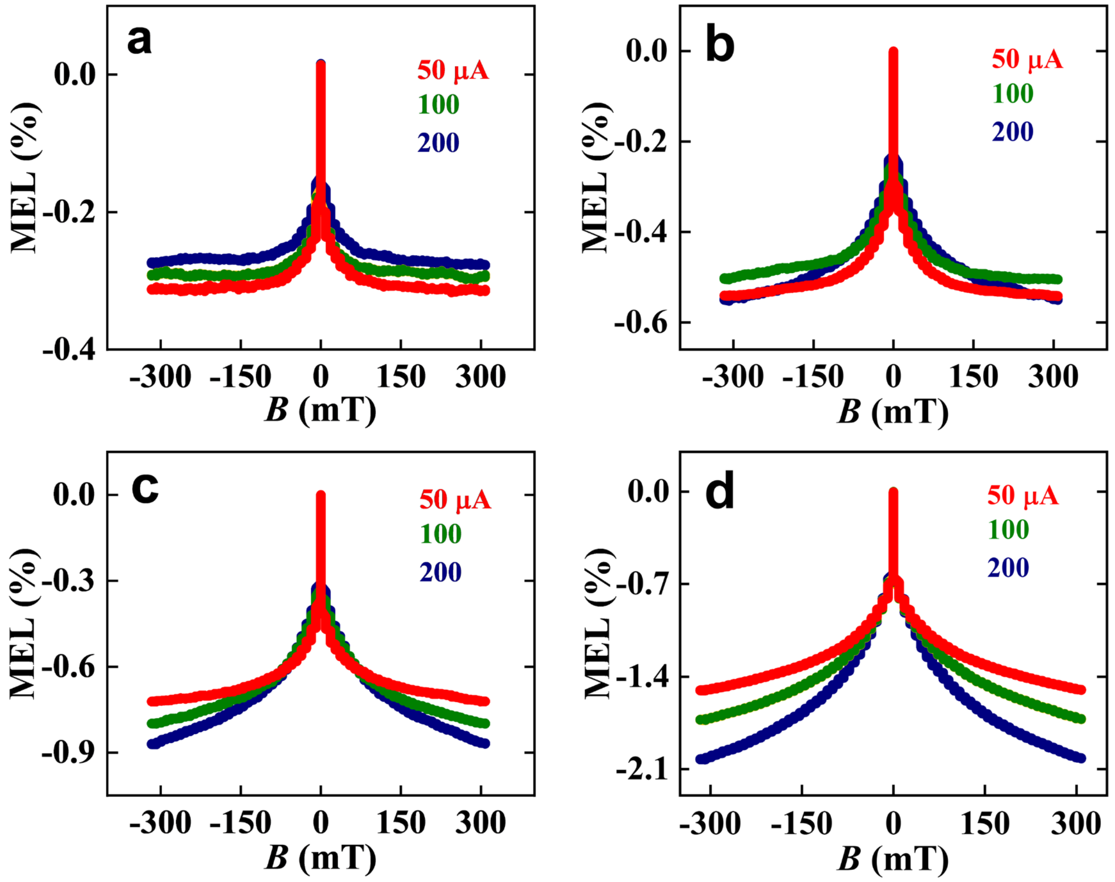

Figure S9. (a-d) The MEL response curves acquired from TBRb molecules in TBRb-doped devices B_1-B_4. All curves were obtained by subtracting the MEL data of non-doped devices A_6 from the MEL data of TBRb-doped devices B_1-B_4.

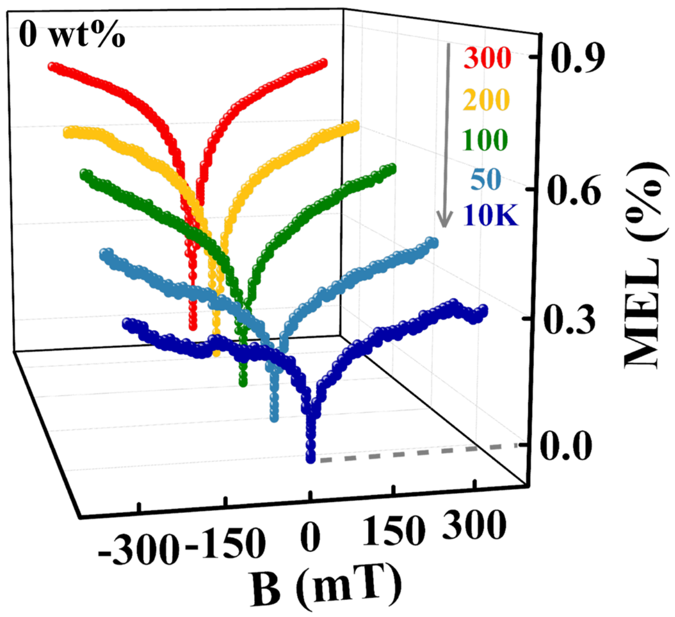

Figure S10. Temperature-dependent MEL traces from the non-doped device A_6 at a bias-current of 100 μA.

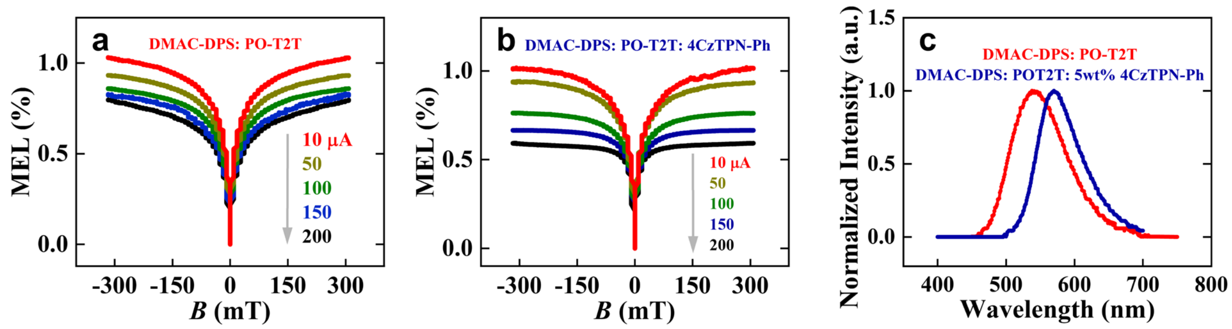

Figure S11. Optical, electrical, and magnetic properties of the DMAC-DPS: PO-T2T exciplex device and the DMAC-DPS: PO-T2T: 5 wt% 4CzTPN-Ph doped device.

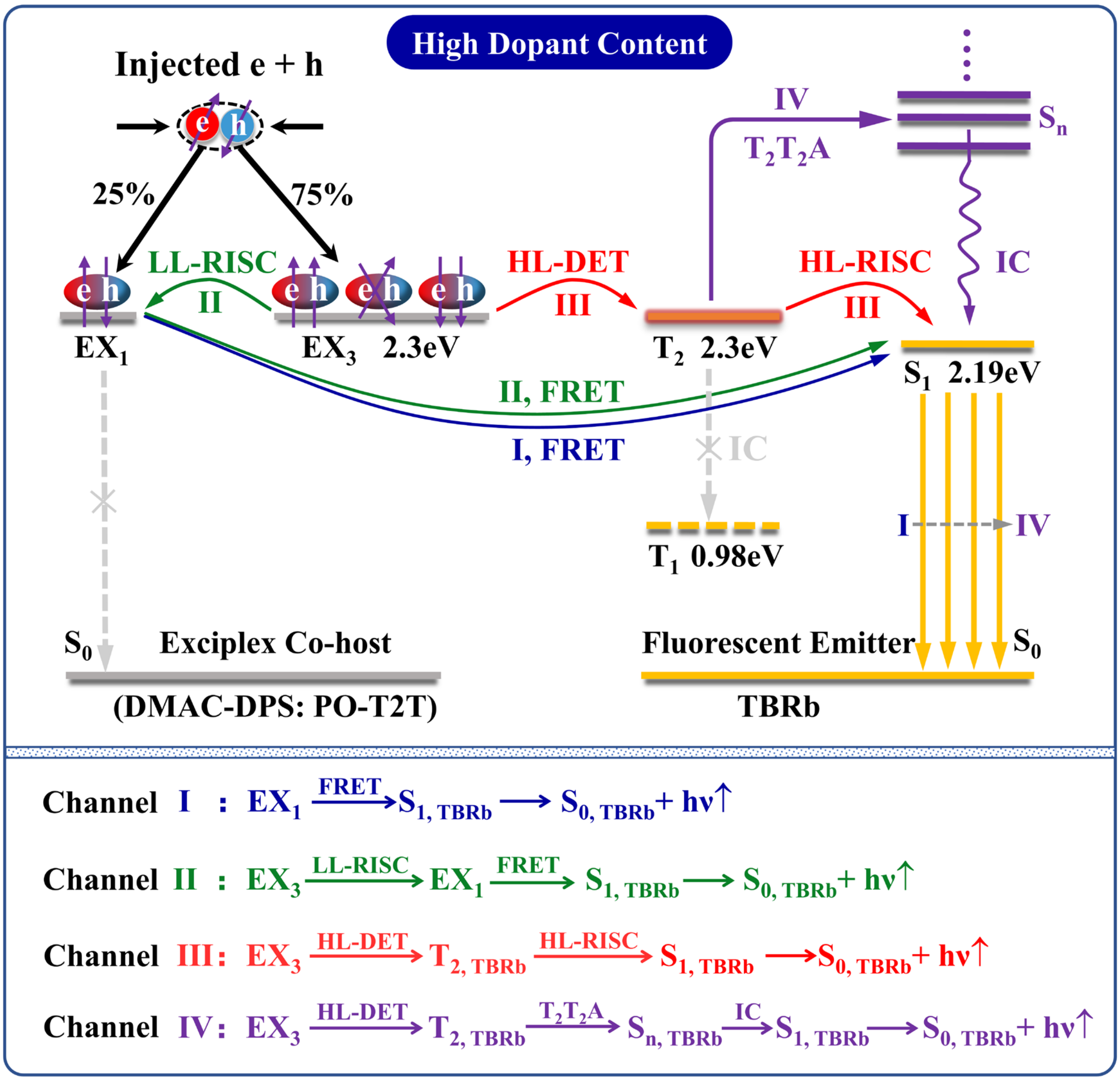

Figure S12. Schematic illustration of energy transfer mechanisms in the TBRb-based devices B_3-B_4 with higher TBRb dopant contents.

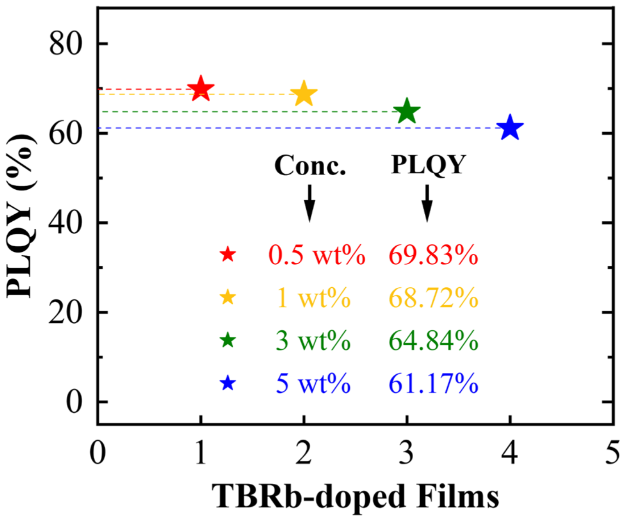

Figure S13. The PLQYs of TBRb-doped films (DMAC-DPS: PO-T2T: x wt% TBRb) with different doping concentrations at room temperature. Conc.: concentration

Reference

[1] C. W. Lin, P. B. Han, S. Xiao, F. L. Qu, J. W. Yao, X. F. Qiao, D.Z. Yang, Y. F. Dai, Q. Sun, D. H. Hu, A. J. Qin, Y. G. Ma, B. Z. Tang, D. G. Ma, *Adv. Funct. Mater.* **2021**, 31, 2106912.

[2] a) C. W. Lin, P. B. Han, F. L. Qu, S. Xiao, Y. Z. Li, D. Xie, X. F. Qiao, D. Z. Yang, Y. F. Dai, Q. Sun, A. J. Qin, B. Z. Tang, D. G. Ma, *Mater. Horiz.* **2022**, 9, 2376; b) S. Xiao, X. F. Qiao, C. W. Lin, Y. Z. Li, S. A. Ying, J. W. Qin, R. D. Guo, L. Wang, Y. G. Ma, D. G. Ma, *Adv. Funct. Mater.* **2022**, 32, 2207123.

[3] a) H. J. Liu, J. J. Zeng, J. J. Guo, H. Nie, Z. J. Zhao, B. Z. Tang, Angew. Chem. Int. Ed. 2018, 57, 9290; b) J. Huang, H. Nie, J. J. Zeng, Z. Y. Zhuang, S. F. Gan, Y. J. Cai, J. J. Guo, S. J. Su, Z. J. Zhao, B. Z. Tang, Angew. Chem. Int. Ed. 2017, 56, 12971

[4] K. H. Kim, C. K. Moon, J. H. Lee, S. Y. Kim, J. J. Kim, *Adv. Mater.* **2014**, 26, 3844.

[5] H. Nakanotani, T. Higuchi, T. Furukawa, K. Masui, K. Morimoto, M. Numata, H. Tanaka, Y. Sagara, T. Yasuda, C. Adachi, *Nat. Commun.* **2014**, 5, 4016.

[6] Y. W. Xu, X. M. Liang, X. H. Zhou, P. S. Yuan, J. D. Zhou, C. Wang, B. B. Li, D. H. Hu, X. F. Qiao, X. F. Jiang, L. L. Liu, S. J. Su, D. G. Ma, Y. G. Ma, *Adv. Mater.* **2019**, 31, 1807388.
